# Effect of Omega-3 Fatty Acid on the Fatty Acid Content of the Erythrocyte Membrane and Proteinuria in Patients with Diabetic Nephropathy

**DOI:** 10.1155/2015/208121

**Published:** 2015-05-18

**Authors:** Su Mi Lee, Seuk Hee Chung, Yongjin Park, Mi Kyoung Park, Young Ki Son, Seong Eun Kim, Won Suk An

**Affiliations:** ^1^Department of Internal Medicine, Dong-A University, 3Ga-1, Dongdaesin-Dong, Seo-Gu, Busan 602-715, Republic of Korea; ^2^Department of Internal Medicine, Ulsan Central Hospital, Ulsan 680-739, Republic of Korea; ^3^Department of Family Medicine, Dong-A University, Busan 602-715, Republic of Korea; ^4^Institute of Medical Science, Dong-A University College of Medicine, Busan 602-714, Republic of Korea

## Abstract

Diabetic nephropathy is the leading cause of end-stage renal disease and is associated with an increased risk of cardiovascular events. Dietary omega-3 fatty acid (FA) has cardioprotective effect and is associated with a slower deterioration of albumin excretion in patients with diabetic nephropathy. In this study, we evaluated the effect of omega-3 FA on proteinuria in diabetic nephropathy patients who are controlling blood pressure (BP) with angiotensin converting enzyme inhibitor (ACEi) or angiotensin II receptor blocker (ARB). In addition, we identified changes in erythrocyte membrane FA contents. A total of 19 patients who were treated with ACEi or ARB for at least 6 months were treated for 12 weeks with omega-3 FA (Omacor, 3 g/day) or a control treatment (olive oil, 3 g/day). Proteinuria levels were unchanged after 12 weeks compared with baseline values in both groups. The erythrocyte membrane contents of omega-3 FA and eicosapentaenoic acid (EPA) were significantly increased, and oleic acid, arachidonic acid : EPA ratio, and omega-6 : omega-3 FA ratio were significantly decreased after 12 weeks compared with the baseline values in the omega-3 FA group. Although omega-3 FA did not appear to alter proteinuria, erythrocyte membrane FA contents, including oleic acid, were altered by omega-3 FA supplementation.

## 1. Introduction

Diabetic nephropathy is the leading cause of end-stage renal disease (ESRD), and the incidence of diabetic nephropathy has been increasing rapidly [1]. Although diabetic nephropathy has been regarded as an irreversible and rapidly progressing disease, progression to kidney failure may be slowed by the use of angiotensin converting enzyme inhibitor (ACEi) or angiotensin II receptor blocker (ARB) [2]. Reducing proteinuria is very important and is the main target for the treatment of diabetic nephropathy [3]. However, there are few options for decreasing proteinuria in diabetic patients who are controlling blood pressure (BP) with ACEi or ARB. Dietary omega-3 fatty acid (FA) is associated with a slower deterioration of albumin excretion in patients with diabetic nephropathy and in model diabetic rats [4–6]. It is not clear whether omega-3 FA has an additional effect on decreasing proteinuria in patients treated with ACEi or ARB.

Diabetic nephropathy is a microvascular complication, and patients with diabetic nephropathy often suffer from accompanying macrovascular complications [7]. Patients with diabetic nephropathy show higher rates of cardiovascular events compared to the general population [8]. Omega-3 FA has been shown to be beneficial in the treatment of cardiovascular disease (CVD), and this cardioprotective effect may be explained by the anti-inflammatory, antioxidative, and antithrombotic abilities of omega-3 FA [9–11]. Several studies have reported that increased intake of omega-3 FA is linked to decreased incidence of atherosclerotic CVD, arrhythmia, and sudden death, although recent meta-analysis did not proved these effects [12–14]. The cardioprotective effects of omega-3 FA are more prominent in diabetics than in nondiabetics [15]. Erythrocyte membrane oleic acid content is significantly higher in patients with acute coronary syndrome than in control subjects [16–18]. However, there are no reports regarding changes of the FA contents of the erythrocyte membrane, including oleic acid, caused by omega-3 FA supplementation in diabetic nephropathy patients with overt proteinuria.

In this study, we hypothesized that omega-3 FA supplementation may decrease proteinuria in patients with BP controlled by ACEi or ARB. In addition, we evaluated the status of erythrocyte membrane FA contents and the effect of omega-3 FA on erythrocyte membrane FA contents, including oleic acid, in diabetic nephropathy patients with overt proteinuria.

## 2. Materials and Methods

### 2.1. Study Design and Patients

We conducted a randomized, double-blind, placebo-controlled study of Dong-A University Nephrology outpatients between June 2009 and October 2010. Nineteen diabetic nephropathy patients, with a proteinuria level > 0.3 g/day and undergoing treatment with ACEi or ARB for at least 6 months, were included. Diabetic nephropathy was defined as diabetic renal disease with proteinuria, with or without elevation of serum creatinine (Cr) levels [19]. Patients matching any of the following criteria were excluded: history of active infection within 3 months; fish oil or omega-3 FA supplementation within 3 months; history of allergies to fish, omega-3 FA, and/or olive oil; history of hospital admission within 3 months; history of bleeding within 3 months; thrombocytopenia; current use of warfarin; an albumin level < 2.5 g/dL; and malignancy and/or liver cirrhosis. Enrolled patients were randomly selected for 12 weeks of treatment with either omega-3 FAs (Omacor, 3 g/day) or a placebo treatment (olive oil, 3 g/day). One gram of Omacor contained 460 mg of eicosapentaenoic acid (EPA) and 380 mg of docosahexaenoic acid (DHA). Randomization was performed using a random number table.

In addition, 32 healthy volunteers were included as normal controls [20]. Healthy volunteers were defined as those with no diabetes mellitus (DM), no urinary abnormalities, and a glomerular filtrate rate (GFR) of more than 60 mL/min/1.73 m^2^. The mean eGFR of healthy volunteers was 87.3 ± 10.6 mL/min/1.73 m^2^. This study was approved by the Dong-A University Hospital Institutional Review Board. Informed consent was obtained from all enrolled patients, and the study was conducted in accordance with the Declaration of Helsinki.

### 2.2. Survey of Food Consumption

Food consumption was surveyed to gather data on the average frequency and portion size at the start of the study and after 12 weeks using a semiquantitative food frequency questionnaire including 121 foods, which was used in the Korean Cancer Research Survey. Three-dimensional food models and full-scale photographs were used to estimate portion size. Nutrient intake was estimated by the Computer Aided Nutritional Analysis Program (Can-Pro 3.0, The Korean Nutrition Society), which includes 1,823 food items.

### 2.3. Laboratory Measurements

Fasting blood samples were obtained, subsequently processed, immediately refrigerated, and stored at −70°C until analysis. Routine laboratory tests were performed, including hemoglobin (Hb), glucose, glycated hemoglobin (HbA1c), blood urea nitrogen (BUN), Cr, GFR, albumin, total cholesterol, triglycerides, high-density lipoprotein cholesterol (HDL), and low-density lipoprotein cholesterol (LDL). Body mass index (BMI) was calculated using weight and height measurements (weight (kg)/height (m^2^)). Proteinuria was measured using random spot urine samples, and urine samples were collected over 24 hours. The levels of urinary neutrophil gelatinase-associated lipocalin (NGAL, R&D systems, Minneapolis, MN, USA) and prostaglandin E_2_ (PGE_2_, R&D systems) were measured by enzyme-linked immunoassay.

### 2.4. Gas Chromatography Procedure

Erythrocyte membrane FA contents were analyzed using gas chromatography (Shimadzu 2010AF, Shimadzu Scientific Instrument, Japan). Isolated erythrocytes were methylated by the addition of boron trifluoride (BF_3_) methanol-benzene for 10 min at 100°C. FA methyl esters were analyzed by gas chromatography with a 100 m SP2560 capillary column (Supelco, Bellefonte, PA, USA). FA was identified by comparison with known standards (GLC-727; Nu-Chek Prep, Elysian, MN, USA). The omega-3 index is a measure of EPA and DHA in erythrocyte membranes. Erythrocyte membrane FA contents were expressed as weight percentages.

### 2.5. Statistical Analysis

Data were expressed as mean ± SD or frequency. Characteristics were analyzed using the Mann-Whitney *U* test or Wilcoxon exact rank sum test for nonparametric data and the Chi-squared test for categorical variables. A *P* value < 0.05 was considered to be statistically significant. All statistical calculations were performed with SPSS software (SPSS version 18.0, Chicago, IL).

## 3. Results

### 3.1. Baseline Characteristics

All 19 enrolled patients completed the trial. The mean age of patients was 60.4 ± 10.7 years, and 63.2% of the study population was male. The mean systolic and diastolic BP readings were 121.1 ± 14.5 mmHg and 72.1 ± 11.8 mmHg, respectively. There were no significant differences between the omega-3 FA group and the placebo group (Table 1).

### 3.2. Comparison of Erythrocyte Membrane FA Contents

We compared the erythrocyte membrane FA contents between healthy volunteers and DM patients (Table 2). When comparing DM patients to healthy controls, the erythrocyte membrane contents of omega-3 FA, DHA, and the omega-3 index were significantly lower, while the erythrocyte membrane contents of oleic acid and the omega-6 FA : omega-3 FA ratio were significantly higher.

### 3.3. Dietary Consumption Data

There were no significant differences in dietary consumption between the omega-3 FA group and the placebo group at baseline. In addition, there were no significant changes in dietary consumption between the omega-3 FA group and the placebo group after 12 weeks compared with baseline values (Table 3).

### 3.4. Changes in Biochemical Data

Glucose, total cholesterol, and LDL levels were significantly decreased in the placebo group after 12 weeks compared with baseline values (*P* = 0.025, *P* = 0.021, and *P* = 0.017, resp.). Analysis of spot urine protein to creatinine ratio, 24 h urine protein, and Cr revealed no alterations after 12 weeks compared with baseline values in both groups. There was a tendency for increased GFR in the omega-3 FA group after 12 weeks compared with baseline values, but this was not statistically significant. In the placebo group, the urinary levels of PGE_2_ and NGAL increased and decreased, respectively, after 12 weeks compared with baseline values, but these changes were not statistically significant. In the omega-3 FA group, urinary levels of PGE_2_ and NGAL were increased and decreased, respectively, after 12 weeks compared with baseline values, but these changes were also not statistically significant (Table 4).

### 3.5. Changes in Erythrocyte Membrane FA Content

The erythrocyte membrane FA contents at baseline showed no significant difference between the 2 groups (Table 5). In the omega-3 FA group, the erythrocyte membrane contents of omega-3 FA and EPA were significantly increased after 12 weeks compared with baseline values (*P* = 0.025, *P* = 0.050, resp.). The erythrocyte membrane contents of oleic acid, arachidonic acid (AA) : EPA ratio, and omega-6 FA : omega-3 FA ratio were significantly decreased after 12 weeks compared with the baseline values in the omega-3 FA group (*P* = 0.036, *P* = 0.012, and *P* = 0.012, resp.). In the placebo group, the erythrocyte membrane contents of palmitoleic acid and AA were significantly increased after 12 weeks compared with baseline values (*P* = 0.046, *P* = 0.046, resp.). The erythrocyte membrane content of oleic acid was decreased in the placebo group after 12 weeks compared with baseline values, but this was not statistically significant.

## 4. Discussion

In this study, we found that omega-3 FA supplementation decreased the erythrocyte membrane oleic acid content in patients with diabetic nephropathy and overt proteinuria. This effect may be induced by elevated erythrocyte levels of omega-3 FA and EPA. Several studies have identified the association between omega-3 FA and diabetes risk. In particular, a recent study has reported that the erythrocyte membrane contents of omega-3 FA were correlated with the risk of type 2 diabetes in a Korean population [21]. They identified that the erythrocyte levels of oleic acid and the omega-6 : omega-3 ratio were positively associated with diabetes risk and that erythrocyte levels of omega-3 FA were negatively associated with diabetes risk. Similar results between the healthy control group and the diabetic nephropathy group were found in the present study. Our previous studies showed that omega-3 FA supplementation decreased erythrocyte membrane oleic acid content in patients treated with hemodialysis and peritoneal dialysis [22, 23]. Therefore, omega-3 FA definitely decreases erythrocyte membrane oleic acid contents in CKD patients regardless of dialysis treatment. The current study is the first to demonstrate the effect of omega-3 FA on the erythrocyte membrane oleic acid content in patients with diabetic nephropathy. It is of note that patients with a high risk of CVD, such as patients with diabetic nephropathy, and those undergoing dialysis have elevated erythrocyte membrane oleic acid contents. We suggest that the possible cardioprotective effect of omega-3 FA supplementation may be related to these changes in erythrocyte membrane FA contents.

Interestingly, we found that the erythrocyte membrane contents of omega-3 FA, EPA, and DHA, in addition to the omega-3 index, tended to increase, and oleic acid tended to decrease, in the control group supplemented with olive oil after 12 weeks compared with baseline values. This result suggests that enough supplementation with olive oil also has a cardioprotective effect in diabetic nephropathy patients. Previous studies have reported that olive oil may affect lipid profiles [24, 25]. In this study, total cholesterol, triglyceride, and LDL levels were decreased in the olive oil group. Thus, the actions of olive oil on lipid metabolism may have potential cardioprotective effects. In addition, omega-3 FA levels were increased after not only omega-3 FA supplementation but also olive oil supplementation. One study has reported that omega-3 FAs could be elevated in olive oil-fed rats [26]. Therefore, olive oil may not have been a good choice for the control treatment in the present study. Another notable result in this study is that although AA levels tended to be lower after omega-3 FA supplementation, there was a significant rise in AA levels after olive oil supplementation. This appears to be a major difference between olive oil and omega-3 FA supplementation. In addition, palmitoleic acid levels were increased by olive oil supplementation. The reason for this may be the increased activity of desaturases, which constitute the rate-limiting step in the process of de novo FA synthesis [27], but this might not be the unique factor affecting this particular finding. Further studies are needed to identify the dose-dependent effects of olive oil on changes in erythrocyte membrane FA.

Proteinuria is a strong predictor of progressive renal dysfunction [28]. It is well recognized that ACEi and ARB are the first-line treatments in diabetic nephropathy with proteinuria [2]. These drugs decrease intraglomerular pressure, systemic arterial pressure, and urinary protein excretion and delay the deterioration in renal function [29–31]. Despite the renoprotective effects of ACEi and ARB, it is difficult to fully halt the progression of proteinuria or reverse existing disease [32, 33]. Therefore, a number of efforts to reduce proteinuria have been attempted. Recently, a growing body of clinical data has demonstrated the antiproteinuric effects of omega-3 FA. Studies in animals have shown that omega-3 FA can be beneficial in retarding the progression of renal failure [34, 35]. Epidemiological studies suggest that omega-3 FA can slow the progression of renal dysfunction [36, 37] and prevent the decline in creatinine clearance in healthy elderly people [38]. In addition, omega-3 FA could slow the progression of proteinuria in patients with type 2 diabetes, but the study in question included patients who were not controlling their BP [4]. In the present study, proteinuria was not significantly decreased in the omega-3 FA supplementation group after 12 weeks compared with baseline values. One possible explanation is that the included patients had already been administrated the maximum dose of ACEi or ARB. This result may suggest that it is difficult to obtain additional antiproteinuric benefits from omega-3 FA. To see the effect of omega-3 FA on proteinuria, further studies separating patients with heavier proteinuria or adding some patients with heavier proteinuria are needed. Alternatively additional studies could estimate the effect of omega-3 FA by using them without ACEi or ARB.

High blood glucose levels lead to diabetic complications, including diabetic nephropathy. Therefore, studies suggest that good glucose control is the most important factor in managing diabetes and its related end-organ damage. The glucose levels were decreased in the olive oil group in this study. These effects may be induced by the actions of olive oil on glucose metabolism and insulin sensitivity [39, 40]. The effect of omega-3 FA on blood glucose control is still uncertain, but it is currently believed that omega-3 FA do not have any adverse effect on this [41, 42]. In this study, glucose levels seemed to be lowered by omega-3 FA supplementation. These results were not related to changes in diet or diabetes medication. Despite decreased glucose levels, HbA1c levels were not significantly altered after omega-3 FA supplementation. This lack of change may be related to the short study period, because HbA1c levels reflect plasma glucose levels over the preceding weeks and months. Further long-term studies are needed to identify the alterations in insulin resistance or glucose levels caused by omega-3 FA supplementation.

NAGL is a 25-kDa lipocalin protein and is produced in epithelial cells and neutrophils in most tissues. It is commonly recognized as a marker of renal tubular damage. Previous studies have reported that NGAL levels may reflect renal impairment and be associated with proteinuria in diabetic patients [43, 44]. Therefore, we hypothesized that omega-3 FA supplementation may affect NGAL levels in patients with diabetic nephropathy. However, our data did not show any statistically significant changes after 12 weeks, despite some apparent decreases in NGAL levels in the omega-3 FA group. One possible explanation is that NGAL levels are highly associated with proteinuria levels [43]. Because our study did not control for the level of proteinuria in both groups, the patients had a wide range of proteinuria extents.

AA is metabolized by 3 major pathways, namely, cyclooxygenase, lipoxygenase, and cytochrome P450, into bioactive eicosanoids. The major Cox-derived eicosanoids are thromboxane and prostaglandins, such as PGE_2_, and have roles in increasing renal blood flow and inhibiting GFR decline in kidney disease [45, 46]. In our study, there were no statistically significant differences in Cr levels and GFR between the 2 study groups, but GFR tended to be higher after omega-3 FA supplementation. PGE_2_ plays an important role as a regulator of pancreatic *β*-cell dysfunction and destruction [47, 48]. Omega-3 FA may protect against *β*-cell dysfunction and destruction and improve insulin sensitivity [49–51]. However, our data did not show any statistically significant changes in PGE_2_ levels after omega-3 FA supplementation. Further large-scale studies are needed.

This study had a number of limitations. First, the poor efficacy of omega-3 FA in preserving renal function and decreasing proteinuria may be due to the smaller number of enrolled patients and the shorter study period, compared to those of other studies. Second, although ethnicity can affect the erythrocyte membrane contents of omega-3 FA, data were only obtained from Korean patients [52, 53].

In summary, although there appears to be no beneficial effect of omega-3 FA on proteinuria, the FA contents of the erythrocyte membrane were significantly altered by omega-3 FA supplementation over 12 weeks in patients with diabetic nephropathy and blood pressure controlled by ACEi or ARB. We conclude that the role of omega-3 FA in altering the FA (including oleic acid) contents of the erythrocyte membrane is not completely clear. Therefore, larger controlled studies evaluating cardiovascular end-points are essential.

## 5. Conclusions

Our results indicate that omega-3 FAs may affect the modification of erythrocyte membrane FA contents in patients with diabetic nephropathy.

## Figures and Tables

**Table 1:** Clinical blood biochemical analyses of the subjects.

	Olive oil (*n* = 8)	Omega-3 FA (*n* = 11)	*P* value^1^
Age (years)	62.0 ± 8.6	59.2 ± 12.2	0.771
Male, *n* (%)	5 (62.5%)	7 (63.6%)	0.960
Systolic BP	121.3 ± 14.6	120.9 ± 15.1	0.966
Diastolic BP	70.0 ± 10.7	73.6 ± 12.9	0.764
BMI	25.0 ± 2.3	25.5 ± 2.8	0.396
Glucose (mg/dL)	158.5 ± 67.6	148.5 ± 64.8	0.680
HbA1c (%)	7.2 ± 1.2	7.2 ± 0.9	0.934
BUN (mg/dL)	19.3 ± 6.1	22.1 ± 6.5	0.320
Creatinine (mg/dL)	1.2 ± 0.2	1.3 ± 0.2	0.116
Estimated GFR	62.7 ± 7.4	55.0 ± 8.0	0.057
Total cholesterol (mg/dL)	162.5 ± 46.8	162.5 ± 36.4	0.967
Triglyceride (mg/dL)	127.0 ± 85.3	142.9 ± 66.9	0.283
HDL (mg/dL)	47.6 ± 13.7	44.7 ± 7.9	0.836
LDL (mg/dL)	88.1 ± 43.4	88.9 ± 33.4	0.934
CRP (mg/dL)	0.15 ± 0.13	0.12 ± 0.13	0.772
Spot urine PCR (g/g)	0.65 ± 0.46	0.79 ± 0.77	0.964
24 hr urine protein (g/g)	0.46 ± 0.44	0.60 ± 0.63	1.000
PGE_2_ (pg/mL)	1494.0 ± 489.8	1308.4 ± 526.5	0.643
NGAL (ng/mL)	30.0 ± 31.2	33.3 ± 60.9	1.000

Data are expressed as means ± SD.

^1^ *P* value for nonparametric Mann-Whitney *U* test comparing baseline data between olive oil group and omega-3 FA group. The difference in frequency was tested using Pearson *χ* ^2^.

BP: blood pressure; BMI: body mass index; HbA1c: glycated hemoglobin; BUN: blood urea nitrogen; GFR: glomerular filtration rate; HDL: high-density lipoprotein cholesterol; LDL: low-density lipoprotein cholesterol; CRP: C-reactive protein; PCR: protein to creatinine ratio; PGE_2_: prostaglandin E_2_; NGAL: neutrophil gelatinase-associated lipocalin.

**Table 2:** Comparison of erythrocyte membrane fatty acids content.

	Control (*n* = 32)	DM (*n* = 19)	*P* value^1^
*Saturated *	39.0 ± 6.1	42.7 ± 7.3	0.081
Myristic	0.57 ± 0.29	0.72 ± 0.32	0.065
Palmitic	24.4 ± 4.0	26.6 ± 4.8	0.159
Stearic	13.9 ± 2.9	15.1 ± 3.5	0.097
Lignoceric	0.23 ± 0.12	0.27 ± 0.17	0.614
*Monounsaturated *	16.2 ± 2.6	17.6 ± 2.9	0.085
Palmitoleic	1.8 ± 1.7	1.2 ± 0.8	0.437
Trans-oleic	1.7 ± 0.7	1.2 ± 0.9	0.036^*^
Oleic	13.9 ± 2.4	15.9 ± 3.0	0.020^*^
*Polyunsaturated *	42.0 ± 7.6	37.4 ± 8.3	0.038^*^
Omega-6	28.9 ± 5.7	27.2 ± 5.7	0.166
Linoleic	13.5 ± 3.0	14.4 ± 4.4	0.785
AA	11.7 ± 3.1	9.4 ± 3.0	0.014^*^
Omega-3	13.1 ± 3.4	10.2 ± 3.9	0.011^*^
Alpha-linolenic	0.35 ± 0.19	0.49 ± 0.21	0.026^*^
EPA	2.0 ± 0.9	1.9 ± 1.1	0.556
DHA	8.3 ± 2.3	6.0 ± 2.2	0.002^*^
Omega-3 index	10.3 ± 2.9	7.9 ± 3.1	0.008^*^
AA/EPA	7.3 ± 4.4	7.1 ± 6.0	0.644
Omega-6/Omega-3	2.3 ± 0.7	3.0 ± 1.1	0.025^*^

Data are expressed as means ± SD.

^1^ *P* value for nonparametric Mann-Whitney *U* test comparing baseline data between control group and DM group.

^*^ *P* value <0.05 (mean values are significantly different from control group).

AA: arachidonic acid; EPA: eicosapentaenoic acid; DHA: docosahexaenoic acid; DM: diabetes mellitus.

**Table 3:** Dietary consumption of foods and nutrients.

	Olive oil (*n* = 8)		Omega-3 FA (*n* = 11)	
	Baseline	12 weeks	Baseline	12 weeks
Kcal (kcal)	1801.3 ± 420.8	1717.2 ± 428.9	1658.4 ± 356.1	1613.4 ± 397.0
Animal protein (g)	29.1 ± 15.9	29.3 ± 17.8	25.2 ± 11.9	22.2 ± 13.3
Vegetable protein (g)	42.5 ± 8.5	39.4 ± 14.6	37.7 ± 12.9	37.1 ± 12.1
Animal lipid (g)	18.2 ± 9.1	21.1 ± 14.8	16.4 ± 6.7	16.7 ± 10.9
Vegetable lipid (g)	16.8 ± 6.4	16.0 ± 9.3	18.8 ± 8.8	17.4 ± 8.1
Carbohydrate (g)	302.8 ± 70.4	278.8 ± 67.1	275.8 ± 51.4	267.3 ± 66.3
Fiber (g)	25.8 ± 8.0	22.7 ± 10.8	24.8 ± 12.1	20.7 ± 8.7
Retinol (*μ*g)	68.5 ± 37.6	89.8 ± 77.3	57.3 ± 35.5	66.5 ± 47.1
Niacin (mg)	16.4 ± 4.1	15.6 ± 5.4	14.9 ± 6.3	13.2 ± 4.3
Vitamin E (mg)	12.3 ± 4.9	11.7 ± 6.0	12.6 ± 6.6	10.5 ± 4.2
Cholesterol (mg)	210.6 ± 108.0	218.8 ± 128.9	192.8 ± 111.6	184.9 ± 111.4

Data are expressed as means ± SD.

The nonparametric Wilcoxon exact rank sum test was used to compare baseline data with 12 weeks data.

**Table 4:** Changes in biochemical data.

	Olive oil (*n* = 8)		Omega-3 FA (*n* = 11)	
	Baseline	12 weeks	Baseline	12 weeks
Systolic BP (mmHg)	121.3 ± 14.6	118.8 ± 14.6	120.9 ± 15.1	123.0 ± 15.7
Diastolic BP (mmHg)	70.0 ± 10.7	70.0 ± 10.7	73.6 ± 12.9	69.0 ± 8.8
BMI (kg/m^2^)	25.0 ± 2.3	25.2 ± 3.1	25.5 ± 2.8	25.4 ± 2.7
Glucose (mg/dL)	158.5 ± 67.6	121.4 ± 50.7^*^	148.5 ± 64.8	128.9 ± 41.8
HbA1c (%)	7.2 ± 1.2	7.0 ± 1.1	7.2 ± 0.9	7.3 ± 1.0
BUN (mg/dL)	19.3 ± 6.1	21.8 ± 6.7	22.1 ± 6.5	22.0 ± 4.8
Creatinine (mg/dL)	1.2 ± 0.2	1.2 ± 0.2	1.3 ± 0.2	1.3 ± 0.3
Estimated GFR (mL/min/1.73 m^2^)	62.7 ± 7.4	63.4 ± 8.4	55.0 ± 8.0	59.2 ± 10.8
Total cholesterol (mg/dL)	162.5 ± 46.8	151.3 ± 42.3^*^	162.5 ± 36.4	161.8 ± 32.7
Triglyceride (mg/dL)	127.0 ± 85.3	113.8 ± 61.3	142.9 ± 66.9	122.9 ± 52.4
HDL (mg/dL)	47.6 ± 13.7	46.3 ± 12.3	44.7 ± 7.9	45.3 ± 11.6
LDL (mg/dL)	88.1 ± 43.4	79.1 ± 39.8^*^	88.9 ± 33.4	88.7 ± 31.3
CRP (mg/dL)	0.15 ± 0.13	0.12 ± 0.10	0.12 ± 0.13	0.18 ± 0.20
Spot urine PCR (g/g)	0.65 ± 0.46	0.66 ± 0.46	0.79 ± 0.77	0.73 ± 0.67
24 hr urine protein (g/g)	0.46 ± 0.44	0.47 ± 0.41	0.60 ± 0.63	0.69 ± 0.65
PGE_2_ (pg/mL)	1494.0 ± 489.8	1355.0 ± 440.1	1308.4 ± 526.5	1548.4 ± 541.8
NGAL (ng/mL)	30.0 ± 31.2	38.5 ± 35.9	33.3 ± 60.9	24.6 ± 18.9

Data are expressed as means ± SD.

The nonparametric Wilcoxon exact rank sum test was used to compare baseline data with 12 weeks data.

^*^ *P* value <0.05 (mean values are significantly different from baseline).

BP: blood pressure; BMI: body mass index; HbA1c: glycated hemoglobin; BUN: blood urea nitrogen; GFR: glomerular filtration rate; HDL: high-density lipoprotein cholesterol; LDL: low-density lipoprotein cholesterol; CRP: C-reactive protein; PCR: protein to creatinine ratio; PGE_2_: prostaglandin E_2_; NGAL: neutrophil gelatinase-associated lipocalin.

**Table 5:** Changes in erythrocyte membrane fatty acids content.

	Olive oil (*n* = 8)		Omega-3 FA (*n* = 11)	
	Baseline	12 weeks	Baseline	12 weeks
*Saturated *	42.5 ± 6.1	37.7 ± 5.3	42.8 ± 8.3	39.8 ± 6.9
Myristic	0.62 ± 0.28	0.44 ± 0.10	0.79 ± 0.34	0.84 ± 0.48
Palmitic	25.2 ± 3.9	23.0 ± 2.9	27.5 ± 5.4	24.6 ± 2.9
Stearic	16.4 ± 3.7	14.0 ± 3.1	14.3 ± 3.1	14.1 ± 5.0
Lignoceric	0.27 ± 0.13	0.25 ± 0.14	0.27 ± 0.20	0.27 ± 0.25
*Monounsaturated *	17.2 ± 3.6	15.1 ± 2.3	17.8 ± 2.5	15.7 ± 1.4
Palmitoleic	1.2 ± 0.9	1.9 ± 1.7^*^	1.2 ± 0.7	1.8 ± 1.6
Transoleic	1.7 ± 1.1	2.0 ± 0.7	0.92 ± 0.61	1.9 ± 0.9
Oleic	15.5 ± 3.7	12.7 ± 1.3	16.2 ± 2.7	13.1 ± 2.2^*^
*Polyunsaturated *	37.4 ± 7.5	43.9 ± 6.6	37.5 ± 9.2	41.5 ± 7.4
Omega-6	26.7 ± 4.6	29.4 ± 5.1	27.6 ± 6.6	23.3 ± 4.7
Linoleic	13.9 ± 4.7	13.2 ± 2.7	14.7 ± 4.4	11.8 ± 3.8
AA	8.9 ± 3.2	11.7 ± 2.9^*^	9.8 ± 2.9	7.9 ± 2.9
Omega-3	10.7 ± 3.1	14.5 ± 2.8	9.8 ± 4.4	18.2 ± 8.4^*^
Alpha-linolenic	0.49 ± 0.24	0.45 ± 0.27	0.49 ± 0.21	0.49 ± 0.23
EPA	1.8 ± 0.6	2.9 ± 1.2	1.9 ± 1.4	4.2 ± 1.5^*^
DHA	6.1 ± 2.0	8.1 ± 1.2	6.0 ± 2.5	7.2 ± 3.2
Omega-3 index	7.9 ± 2.3	11.0 ± 2.1	7.9 ± 3.7	11.3 ± 4.6
AA/EPA	5.4 ± 3.0	4.7 ± 2.2	8.3 ± 7.4	1.9 ± 0.4^*^
Omega-6/Omega-3	2.7 ± 0.7	2.1 ± 0.5^*^	3.2 ± 1.3	1.5 ± 0.7^*^

Data are expressed as means ± SD.

The nonparametric Wilcoxon exact rank sum test was used to compare baseline data with 12 weeks data.

^*^ *P* value <0.05 (mean values are significantly different from baseline).

AA: arachidonic acid; EPA: eicosapentaenoic acid; DHA: docosahexaenoic acid.
